# Thiol-Functionalized Ethylene Periodic Mesoporous Organosilica as an Efficient Scavenger for Palladium: Confirming the Homogeneous Character of the Suzuki Reaction

**DOI:** 10.3390/ma13030623

**Published:** 2020-01-30

**Authors:** María I. López, Dolores Esquivel, César Jiménez-Sanchidrián, Pascal Van Der Voort, Francisco J. Romero-Salguero

**Affiliations:** 1 Departamento de Mecánica, Área de Ciencia de los Materiales e Ingeniería Metalúrgica, Escuela Politécnica Superior de Córdoba, Universidad de Córdoba, Campus de Rabanales, Edificio Leonardo Da Vinci, E-14071 Córdoba, Spain; 2 Departamento de Química Orgánica, Instituto Universitario de Nanoquímica, Facultad de Ciencias, Universidad de Córdoba, Campus de Rabanales, Edificio Marie Curie, E-14071 Córdoba, Spain; qo1jisac@uco.es (C.J.-S.); qo2rosaf@uco.es (F.J.R.-S.); 3 Department of Chemistry, Ghent University, Krijgslaan 281-S3, B-9000 Ghent, Belgium; Pascal.VanDerVoort@UGent.be

**Keywords:** periodic mesoporous organosilicas, thiol-functionalized organosilicas, Pd-supported catalysts, Suzuki cross-coupling reaction, Pd scavenger

## Abstract

This work describes the synthesis of thiol-functionalized periodic mesoporous organosilicas (PMOs) prepared using the precursor 1-thiol-1,2-bis(triethoxysilyl)ethane, alone or mixed with 1,2-bis(triethoxysilyl)ethane. The thiol groups incorporated into the structure were found to be efficient for palladium binding. This has allowed these materials to be used as catalysts in the Suzuki cross-coupling reaction of bromobenzene and phenylboronic acid. Their performance has been compared to palladium-supported periodic mesoporous (organo)silicas and important differences have been observed between them. The use of different heterogeneity tests, such as hot filtration test and poisoning experiments, has provided a deep insight into the reaction mechanism and has confirmed that the reaction occurs in the homogeneous phase following a “release and catch” mechanism. Furthermore, the thiol-functionalized periodic mesoporous organosilica, synthesized using only 1-thiol-1,2-bis(triethoxysilyl)ethane as a precursor, has proven to be an efficient palladium scavenger.

## 1. Introduction

Periodic mesoporous organosilicas (PMOs) are a particular type of mesoporous organosilicas that are prepared from bridged silsesquioxanes [1,2,3]. Thus, alternate organic and inorganic fragments are present in their structure. Unlike organosilicas synthesized by grafting or co-condensation, PMOs exhibit some clear advantages, such as the homogeneous distribution of organic moieties, absence of pore blocking and high loading of organic units [4,5]. The organic bridges in these materials can be of very different nature and complexity. Even the simplest ones impart some interesting features to these materials, such as enhanced hydrophobicity and increased mechanical and hydrothermal stabilities in comparison with their silica counterparts.

Functional groups can be incorporated into PMOs either by direct synthesis from proper precursors or by post-functionalization of reactive bridges [4]. Usually, only short and/or relatively rigid bridged precursors can lead to ordered structures, giving rise to 100% PMO materials. Large and/or flexible bridged precursors usually need to be co-condensed with other simple precursors, such as TEOS or bis(triethoxysilyl)ethane in order to obtain periodic materials.

Both routes have been used to prepare sulfur-containing PMOs, which are interesting materials for the coordination of heavy and noble metals. Thus, bridged precursors such as bis(3-(triethoxysilyl)propyl)disulfide and bis(3-(triethoxysilyl)propyl)tetrasulfide have been incorporated in PMOs and used for the adsorption of Hg^2+^ [6,7,8,9]. Also, the precursor (3-mercaptopropyl)triethoxysilane has been profusely employed for introducing thiol groups into mesoporous (organo)silicas [10,11,12]. In this context, an option that allows thiol groups to be obtained without involving the use of grafting or co-condensation processes is the use of the precursor 1-thiol-1,2-bis(triethoxysilyl)ethane, synthesized by Van Der Voort et al. by thiol acid-ene chemistry [13]. This yielded a 100% thiol-functionalized PMO, which proved to have a high adsorption capacity and selectivity towards mercury ions [14].

In particular, materials with organic ligands containing sulfur can stabilize palladium complexes and nanoparticles in order to preserve their catalytic activity. This makes these type of materials promising catalysts in carbon–carbon bond-forming reactions, such as Suzuki, Negishi, Kumada and Stille cross-coupling reactions, which are processes with important applications in organic chemistry [15,16,17,18]. To catalyze the aforementioned cross-coupling reactions, palladium-based catalysts are used in most cases because they have good catalytic activity and selectivity [19]. Among these transformations, the Suzuki cross-coupling reaction, which occurs by reaction of aryl halides with aryl boronic acids, stands out for being an extremely useful and highly efficient synthetic method for preparing biaryls, which have extensive applications in the agrochemical and pharmaceutical industries [20,21,22].

Typically, the Suzuki cross-coupling reaction is catalyzed by soluble palladium complexes, such as phosphorus or nitrogen-based complexes or “ligandless” palladium catalysts [23,24,25,26]. Although homogeneous catalysts frequently exhibit very high activity and selectivity, they usually have important drawbacks, as they normally are not reusable and also, the reaction products are frequently contaminated by palladium [27,28].

However, despite the numerous published articles in which it is stated that the supported palladium catalysts used in the Suzuki cross-coupling reaction are heterogeneous, when more stringent tests are performed, it is most often demonstrated that the true active species come from leached metals [26,29,30,31,32,33]. In fact, Jones et al. published, in 2006, a rigorous and thorough review in which they asserted that there is no direct evidence for a truly heterogeneous palladium Heck or Suzuki coupling catalyst-based palladium nanoparticle- or macroparticle-catalyzed turnover [26]. In this review, it was shown that many authors presuppose that the form of palladium added to the reactor is the active catalyst, without conducting a study of the nature of the truly catalytic species. In other cases, the authors make use of several tests to ascertain whether the active species is leached palladium or solid-phase palladium, but the results are misunderstood because many of the tests used to assess the heterogeneity of catalyzed processes and the recyclability of the catalysts (for example, hot filtration test and the comparison of the final yield after catalyst reuse) provide inconclusive results for palladium-catalyzed coupling reactions. This difficulty in interpreting the results is mainly due to the following reasons: i) traces of soluble palladium metal can act as highly active catalysts [34,35,36,37,38], ii) soluble palladium can be deactivated in numerous ways (for example, redeposition on supports and overcoordination by strongly binding ligands) [39] and iii) palladium can be found in different solid phases or in solution [40,41]. Therefore, commonly used heterogeneity tests have to be interpreted with caution and must be supported through the use of other methods.

Herein, we report the synthesis of thiol-functionalized periodic mesoporous organosilicas (PMOs) using the precursor 1-thiol-1,2-bis(triethoxysilyl)ethane, alone or mixed with 1,2-bis(triethoxysilyl)ethane. After the incorporation of palladium, these materials, as well as palladium-supported periodic mesoporous (organo)silicas, have been evaluated as catalysts in the Suzuki cross-coupling reaction. The results obtained, together with the application of two standard techniques (hot filtration test and catalyst poisoning test), showed that the mechanism that follows this reaction is the so-called “release and catch” [42], which confirms the homogeneous nature of this reaction.

## 2. Materials and Methods 

### 2.1. Reagents and Materials

The Grubbs´ first-generation catalyst (PCy_3_)_2_Cl_2_Ru = CHPh) (97%), vinyltriethoxysilane (97%), 2,2-dimethoxy-2-phenylacetophenone (99%), thioacetic acid (96%), propylamine (98%), pluronic P123, palladium (II) acetate (98%), palladium (II) acetylacetonate (99%), bromobenzene (99%) and phenylboronic acid (95%) were supplied by Aldrich (St. Louis, MO, USA). KCl (≥99.5%), HCl (37%), ethanol (≥96%, denaturated) and tetrahydrofuran (anhydrous) were provided by Carl-Roth (Karlsruhe, Germanuy). Also, K_2_CO_3_ (99%, Panreac, Chicago, IL, USA) and dodecane (95%, Fluka, Buchs, Switzerland) were used. All these chemicals were used without further purification.

### 2.2. Synthesis of the Catalysts

Precursor 1-thiol-1,2-bis(triethoxysilyl)ethane was synthesized by a thiol acid-ene reaction between the E-diastereoisomer of bis(triethoxysilyl)ethene [43] and thioacetic acid under photochemical conditions [13]. In this work, two thiol-functionalized ethylene-bridged PMOs were synthesized following a similar procedure to that described in [44]. Typically, P123 surfactant (0.42 g) and KCl (2.1 g) were added to a solution of HCl (2.1 mL) in water (14.76 mL). The resulting mixture was vigorously stirred overnight at 45 °C. 1-Thiol-1,2-bis(triethoxysilyl)ethane or an equimolar mixture of 1-thiol-1,2-bis(triethoxysilyl)ethane and bis(triethoxysilyl)ethane was then added to the resulting clear solution. The reactant molar ratio Si:KCl:H_2_O:HCl:P123 was 1.0:6.9:58.5:4.9:0.013. The synthesis mixture was stirred at 45 °C for 24 h and subsequently aged at 100 °C for 24 h under static conditions. A white solid was recovered by filtration and it was thoroughly washed with water. The surfactant was removed by refluxing the as-synthesized material (0.5 g) in a solution of 4 mL of HCl in 150 mL of ethanol for 6 h. This process was repeated twice and then the solid was filtered, washed with ethanol and dried under vacuum at 120 °C. The material synthesized using only 1-thiol-1,2-bis(triethoxysilyl)ethane as a precursor was named as SH-PMO, while that obtained using an equimolar mixture of the two precursors was designated as SH-E-PMO.

Once both materials were synthesized, the adsorption of palladium was carried out following a similar procedure to that described by Crudden et al. [45]. Briefly, 115 mg (0.51 mmol) of palladium (II) acetate, Pd(OAc)_2_, was dissolved in 75 mL of dry tetrahydrofuran. This was stirred under an argon atmosphere for 15 min in order to achieve its complete dissolution. The amount of material containing 1.03 mmol of S was then added to the solution and the mixture was stirred under argon for 1 h at room temperature. Finally, the solid was filtered and dried under vacuum at 120 °C. This procedure was performed on SH-PMO and SH-E-PMO materials. Thereby, the catalysts named as Pd-SH-PMO and Pd-SH-E-PMO, respectively, were obtained. A portion of these materials was treated with H_2_ at 200°C for 1 h in order to obtain the materials designated as Pd-SH-PMO(H_2_) and Pd-SH-E-PMO(H_2_).

For comparison, ethylene- and phenylene-bridged PMOs (E-PMO and Ph-PMO, respectively), as well as a periodic mesoporous silica (PMS), synthesized by using Brij-76 as surfactant under acidic conditions according to previously reported procedures [46], were also used as supports. In this case, palladium was deposited in these mesoporous materials by impregnation to incipient wetness [47]. Briefly, 5 mL of acetone containing 0.03 g of palladium (II) acetylacetonate, Pd(acac)_2_, were added to 1.0 g of support to obtain a metal loading of 1.0 wt% Pd. The resulting suspension was stirred in a rotavapor at 40 °C for 24 h to remove the solvent. The impregnated materials were dried at 100 °C under vacuum, thus obtaining the materials designed as Pd^2+^/E-PMO, Pd^2+^/Ph-PMO and Pd^2+^/PMS. They were reduced with H_2_ at 200 °C for 1 h in order to obtain the materials named as Pd^0^/E-PMO, Pd^0^/Ph-PMO and Pd^0^/PMS, respectively. 

### 2.3. Characterization of the Catalysts

Powder X-ray diffraction (PXRD) patterns were recorded using an ARL X´TRA (Thermo Scientific, Waltham, MA, USA) powder diffractometer with Cu-Kα radiation of 0.15418 nm wavelength and a solid-state detector. Transmission electron microscopy (TEM) images were obtained on a JEOL JEM 1400 instrument (Tokio, Japan) on samples supported on copper grids with carbon coating. Nitrogen adsorption–desorption experiments were performed using a Micromeritics TriStar 3000 analyzer (Norcross, GA, USA) at −196 °C. Prior to measurements, the samples were outgassed at 120 °C for 24 h. The specific surface area was calculated applying the Brunauer–Emmett–Teller (BET) method over a relative pressure (P/P_0_) range of 0.05–0.15, and the pore size distribution was obtained by analysis of the adsorption branch of the isotherms using the Barret–Joyner–Halenda (BJH) method. Diffuse reflectance infrared Fourier transform spectroscopy (DRIFTS) measurements were carried out on a Thermo Nicolet 6700 FT-IR spectrometer (Waltham, MA, USA) equipped with a N_2_-cooled MCT-A (mercury-cadmium-tellurium) detector and a KBr beam splitter. Elemental compositions were determined on a Thermo Flash 2000 analyzer (Waltham, MA, USA) by using V_2_O_5_ as catalyst. The atomic absorption spectrometry (AAS) measurements were recorded on a Varian SpectrAA 220FS spectrometer (Palo Alto, CA, USA). Raman spectra were acquired with a Renishaw Raman instrument (inVia Raman Microscope, Wotton-under-Edge, UK) by excitation with green laser light (532 nm) and a grating of 1800 lines/mm. In order to improve the signal-to-noise ratio, a total of 20 scans per spectrum were recorded. X-ray photoelectron spectroscopy (XPS) spectra were recorded with a SPECS Phoibos HAS 3500 150 MCD (Berlin, Germany). The residual pressure in the analysis chamber was 5·10^−9^ Pa. Accurate binding energies were determined with respect to the position of the Si 2p peak at 103.4 eV. The peaks were decomposed using a least-squares fitting routine (Casa XPS software version 2.3.15, Teignmouth, UK) with a Gauss/Lorenz ratio of 70/30.

### 2.4. Catalytic Activity

#### 2.4.1. General Procedure

The catalytic activity of the materials was evaluated in the Suzuki cross-coupling reaction. This reaction was conducted according to a modified version of the procedure described in [48]. Thus, the catalyst (2.5 mol% Pd) was pretreated overnight at 120 °C and then added to a mixture of bromobenzene (0.5 mmol), phenylboronic acid (0.75 mmol), K_2_CO_3_ (1.0 mmol), dodecane (0.5 mmol) as internal standard and EtOH (5.0 mL) in a 10 mL round-bottom flask. The resulting mixture was then stirred at 80 °C under air. Samples were collected at regular intervals and then were analyzed by gas chromatography by using a VF-5ms 30 m × 0.25 mm ID capillary column. Reaction products were identified by the use of standards and quantified with dodecane as an internal standard by using an FID detector. Biphenyl was the only product detected.

#### 2.4.2. Hot Filtration Tests

For hot filtration tests, the same process described in Section 2.4.1. was followed and after a certain time—usually 5 or 15 min—the reaction mixture was filtered off at 80 °C using a syringe with a 0.20 µm nylon membrane filter, with the aim of eliminating all catalyst particles. The clear solution obtained was introduced into another round-bottom flask at 80 °C under air. The conversion was monitored by gas chromatography.

#### 2.4.3. Catalyst Poisoning Tests

For the catalyst poisoning test, SH-PMO was added at the beginning or during the reaction to give a S:Pd ratio of 1.15.

## 3. Results

### 3.1. Characterization of the Catalysts

The X-ray diffraction (XRD) patterns of samples SH-PMO and SH-E-PMO, as well as the corresponding materials with palladium incorporated, exhibited an intense (100) peak and broad and short second-order (110) and (200) peaks at higher diffraction angles, indicative of materials with 2D hexagonal (p6mm) mesostructures (Figure S1 and Table 1) [13]. These results showed that the well-ordered two-dimensional hexagonal structure was conserved after the incorporation of palladium. However, the peaks were slightly shifted towards higher diffraction angles, thus indicating a structural contraction.

The composition of all samples is given in Table 2. The incorporation of palladium through adsorption was confirmed by atomic absorption spectrometry (AAS). Sulfur-containing PMO materials resulted in S to Pd ratios in the same range—close to 8. Although it is a very high value, it should be considered that S is present in SH-PMO and SH-E-PMO both as thiol (-SH) and disulfide (-S-S-) groups. In addition, these groups are eventually distributed in the pore walls and so they might be inaccessible to Pd species. Sulfur groups are considered to be essential since an ethylene-bridged PMO material did not adsorb Pd under the same conditions.

N_2_ adsorption–desorption isotherms were of type IV, with a step at a relative pressure of 0.4–0.7, i.e., typical of mesoporous solids (Figure S2). All materials exhibited a high surface area (Table 1). The average pore diameter and the narrow pore size distribution confirmed the presence of pores in the mesopore range (Figure S3). It is noteworthy that the Pd adsorption in both S-containing supports caused an important decrease in surface area, pore volume and pore diameter. For comparison, the ethylene-bridged PMO preserved its surface area after the incorporation of Pd by impregnation (see Supplementary Information, Table S1).

**Figure 1:**
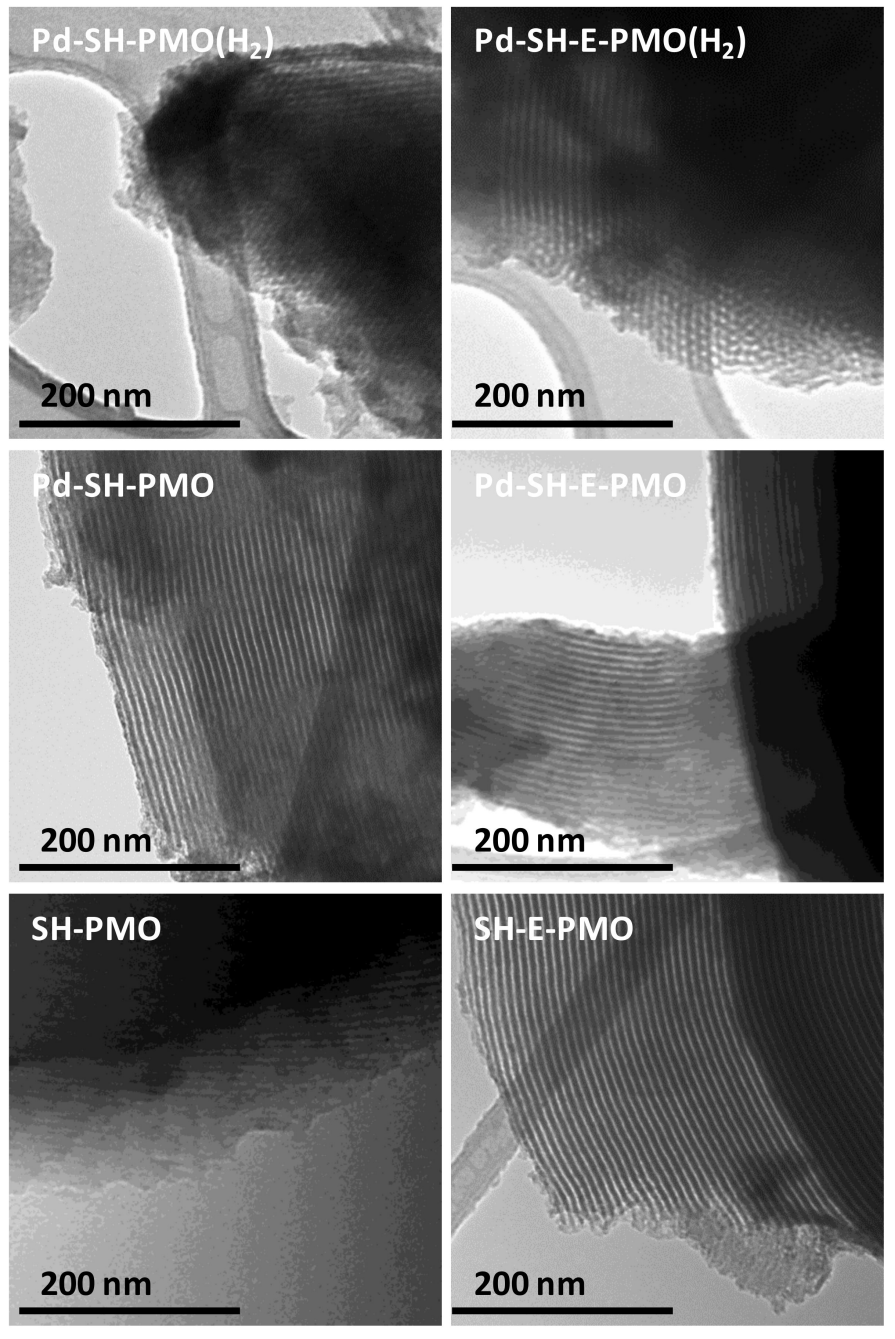
Representative TEM images of SH-PMO and SH-E-PMO (with and without palladium), showing the front and side projections of the mesostructure.

Transmission electron microscopy (TEM) images of these materials (Figure 1), in which the pore openings with hexagonal array and the channel structure of the materials can clearly be seen, were consistent with the XRD results. Pd particles were not observed, even after H_2_ treatment, which suggests that Pd is atomically dispersed by interaction with thiol groups.

**Figure 2:**
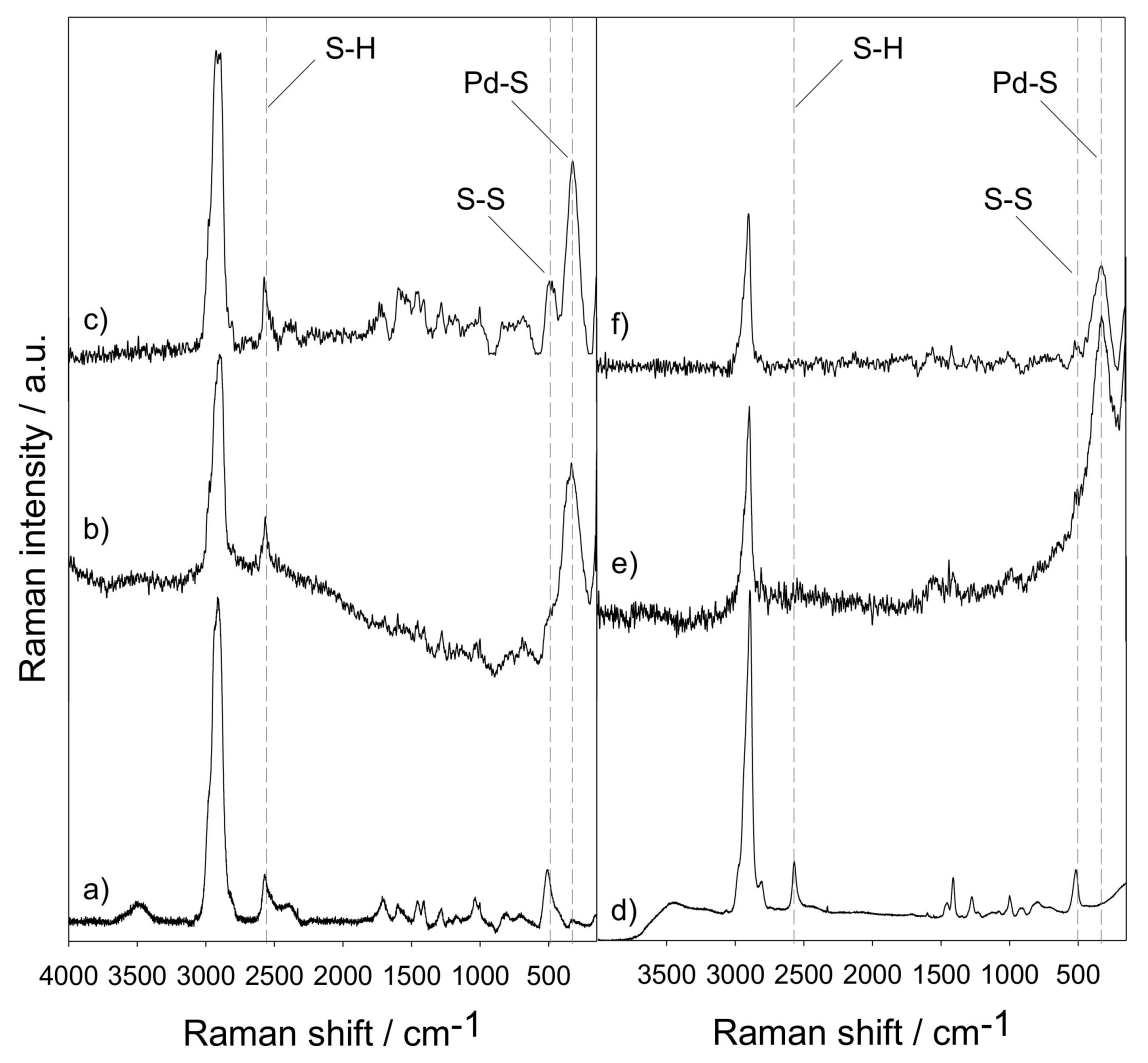
Raman spectra of SH-PMO and SH-E-PMO (with and without palladium): (**a**) SH-PMO, (**b**) Pd-SH-PMO, (**c**) Pd-SH-PMO(H_2_), (**d**) SH-E-PMO, (**e**) Pd-SH-E-PMO, (**f**) Pd-SH-E-PMO(H_2_).

The existence of organic bridges in the structure was proven by diffuse reflectance infrared Fourier transform spectroscopy (DRIFTS). Thus, bands at 1276, 1411, 2900 and 2978 cm^−1^ were assigned to C-H vibrations of the ethylene bridges (Figure S4) [49]. In addition, a tiny peak from S-H stretching vibrations can be observed at ca. 2560 cm^−1^ in SH-PMO and SH-E-PMO materials. The presence of S-H groups was clearly confirmed by Raman spectroscopy (Figure 2). Thus, the band at 2570 cm^−1^ was assigned to S-H stretching vibrations. Moreover, the band at 520 cm^−1^ corresponded to S-S vibrations, which indicates that sulfur is present both in thiol and in disulfide groups. These latter are formed by the oxidation of two thiol groups. The reaction happens spontaneously and is unavoidable in an environment containing oxygen. Furthermore, this technique allowed Pd-S bonds to be detected as revealed by the presence of a band at 330 cm^−1^ [50].

### 3.2. Catalytic Activity

**Scheme 1:**
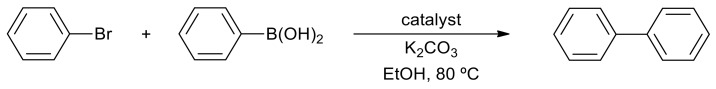
Suzuki cross-coupling reaction between bromobenzene and phenylboronic acid.

Pd containing PMOs were tested as catalysts for the Suzuki cross-coupling reaction between phenylboronic acid and bromobenzene (Scheme 1).

**Figure 3:**
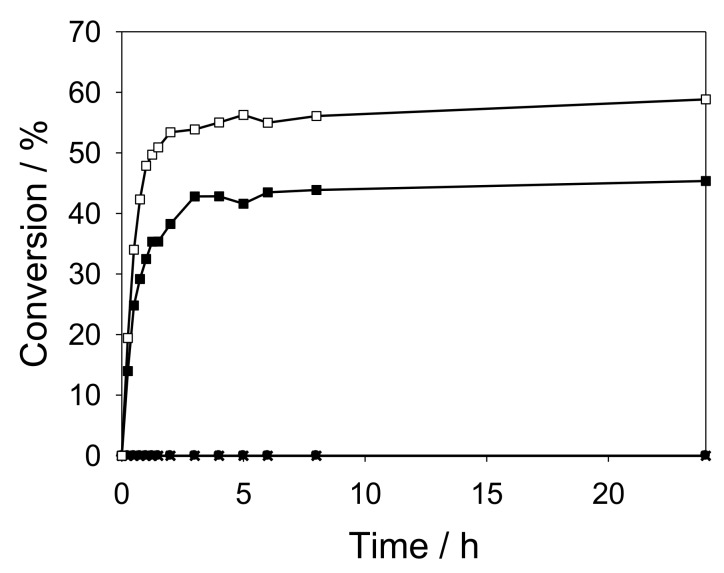
Overall conversion (mol%) in the Suzuki cross-coupling reaction of bromobenzene and phenylboronic acid: Pd-SH-PMO (●), Pd-SH-PMO(H_2_) (○), Pd-SH-E-PMO (■), Pd-SH-E-PMO(H_2_) (□), blank (x). Note: The conversion was null in the blank reaction and when Pd-SH-PMO and Pd-SH-PMO(H_2_) catalysts were used.

Surprisingly, material Pd-SH-PMO was completely inactive, whereas material Pd-SH-E-PMO gave a moderate activity (Figure 3).

As can be seen in Table 2, the S/Pd atomic ratios in Pd-SH-PMO, Pd-SH-PMO(H_2_), Pd-SH-E-PMO and Pd-SH-E-PMO(H_2_) were similar. However, the first two materials had much higher sulfur densities, defined as sulfur atoms per square nanometer of material, practically tripling those of Pd-SH-E-PMO and Pd-SH-E-PMO(H_2_), respectively. A plausible reason for the absence of activity shown by these two materials is that the large number of available sulfur sites rapidly sequestres leached Pd species. This hypothesis suggests that this reaction is catalyzed in solution-phase.

**Figure 4:**
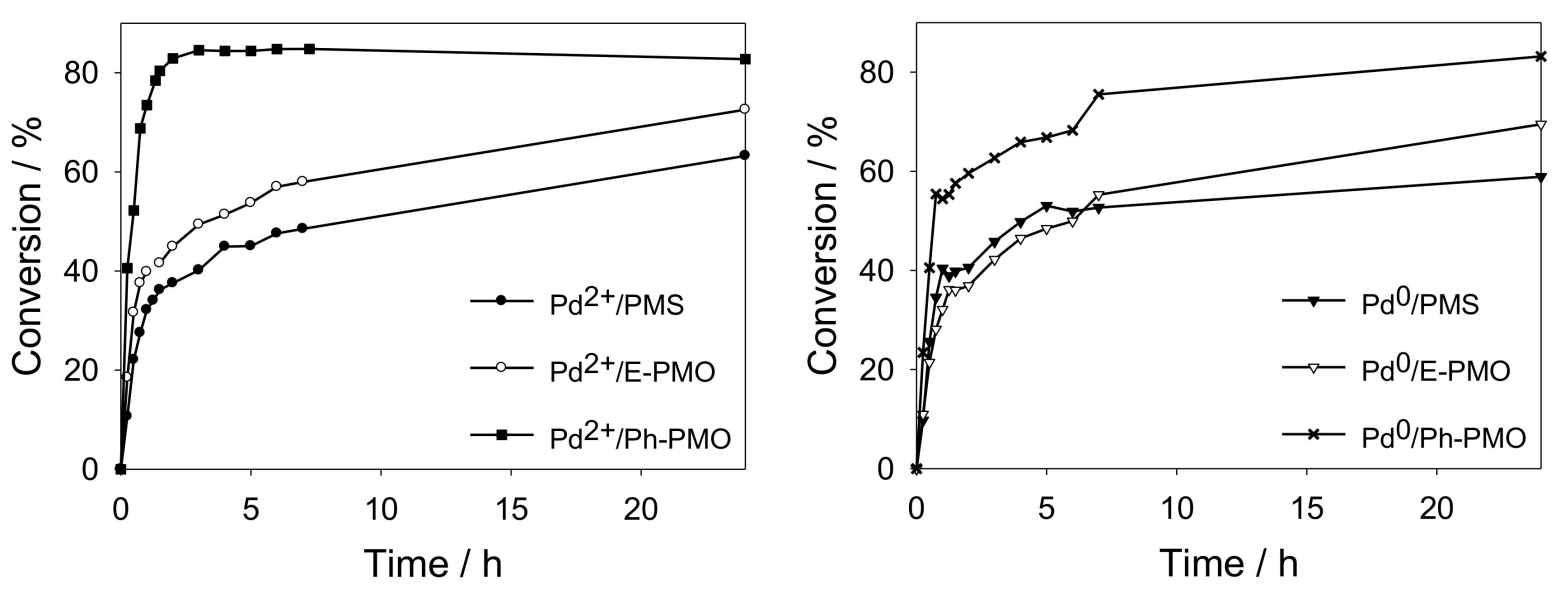
Overall conversion (mol%) in the Suzuki cross-coupling reaction of bromobenzene and phenylboronic acid with Pd^2+^ and Pd-supported periodic mesoporous (organo)silicas as catalysts.

In order to compare their activities with other non-functionalized Pd-containing PMOs, three catalysts consisting of 1 wt% Pd on three supports, i.e., periodic mesoporous silica (Pd^2+^/PMS), ethylene-bridged periodic mesoporous organosilica (Pd^2+^/E-PMO) and phenylene-bridged periodic mesoporous organosilica (Pd^2+^/Ph-PMO), were tested under the same reaction conditions (Figure 4, left). The last one was the most active and provided a conversion above 80% in 1.5 h. Pd^2+^/E-PMO and Pd^2+^/PMS gave rise to a lower activity, in particular, the latter one. On the other hand, these three materials were reduced under a hydrogen stream at 200 °C. Under these conditions, Pd^2+^ is reduced to Pd^0^ [47]. The reduced catalysts were also used for the Suzuki reaction and exhibited a similar conversion to the unreduced catalysts, except for the Ph-PMO support, which presented a slightly lower reaction rate even though after 24 h it gave the same conversion (Figure 4, right). As indicated above, the same reduction treatment was applied to materials Pd-SH-PMO and Pd-SH-E-PMO. However, the use of different techniques, such as TEM (Figure 2) and XPS (Figure S5), provided no evidence of the reduction of Pd^2+^ to Pd^0^ in these materials. The hydrogen-treated Pd-SH-PMO material remained completely inactive. On the contrary, the hydrogen-treated Pd-SH-E-PMO material provided a higher conversion than the untreated material (Figure 3). This increase in catalytic activity may be due to the fact that the treatment with hydrogen at high temperature causes a distortion in the structure that makes palladium passes more easily to solution and, as a consequence, the conversion obtained with this material is higher.

With the purpose of testing the homogeneous/heterogeneous character of the Suzuki cross-coupling reaction of bromobenzene and phenylboronic acid, two standard techniques, i.e., hot filtration test and catalyst poisoning test [51], were carried out.

**Figure 5:**
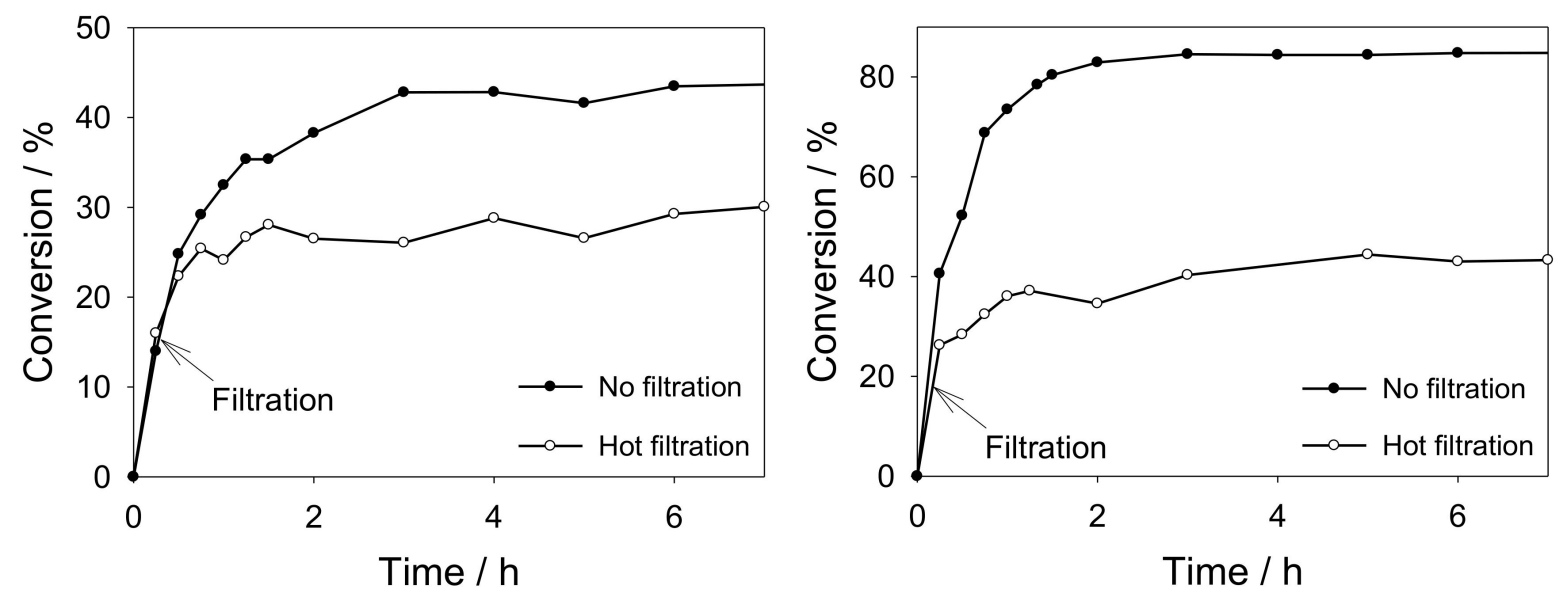
Application of the hot filtration test to Pd-SH-E-PMO (left) and Pd^2+^/Ph-PMO (right) catalyzed Suzuki cross-coupling reaction of bromobenzene and phenylboronic acid. Hot filtration was carried out at 15 min in the first case and at 5 min in the second case.

In the hot filtration test, also called the split test, the solid catalyst is filtered out of the reaction and the filtrate is tested for catalytic activity. The observation of activity in the filtrate is indicative of soluble palladium species present in solution. Figure 5 depicts the hot filtration test for two catalysts: Pd-SH-E-PMO and Pd^2+^/Ph-PMO. As can be observed, after filtration, the reaction did not progress and the conversion was lower than in the presence of the catalyst. However, although the lack of activity in the filtrate is usually interpreted as proof that the reaction takes place heterogeneously, this affirmation cannot be made with palladium-catalyzed reactions without confirmation by other tests [52]. This may be due to several reasons. First, because if there are particles of palladium present in the solution, they can redeposit on the support prior to or during filtration [26]. On the other hand, disruptions, for instance hot filtration, can induce the deactivation of small amounts of active metal, which can lead to the wrong conclusion that there are not active soluble catalytic species before the filtration [26]. It could also happen that, as Suzuki cross-coupling reactions employ slightly soluble reagents (such as boronic acids), they may be removed during filtration [45]. To ensure that this latter did not occur in our case, the same initial amounts of reactants were added to the reaction medium after 4 h. Thereby, the absence of activity confirmed that it was not due to the elimination of some of the reactants during the catalyst filtration.

**Figure 6:**
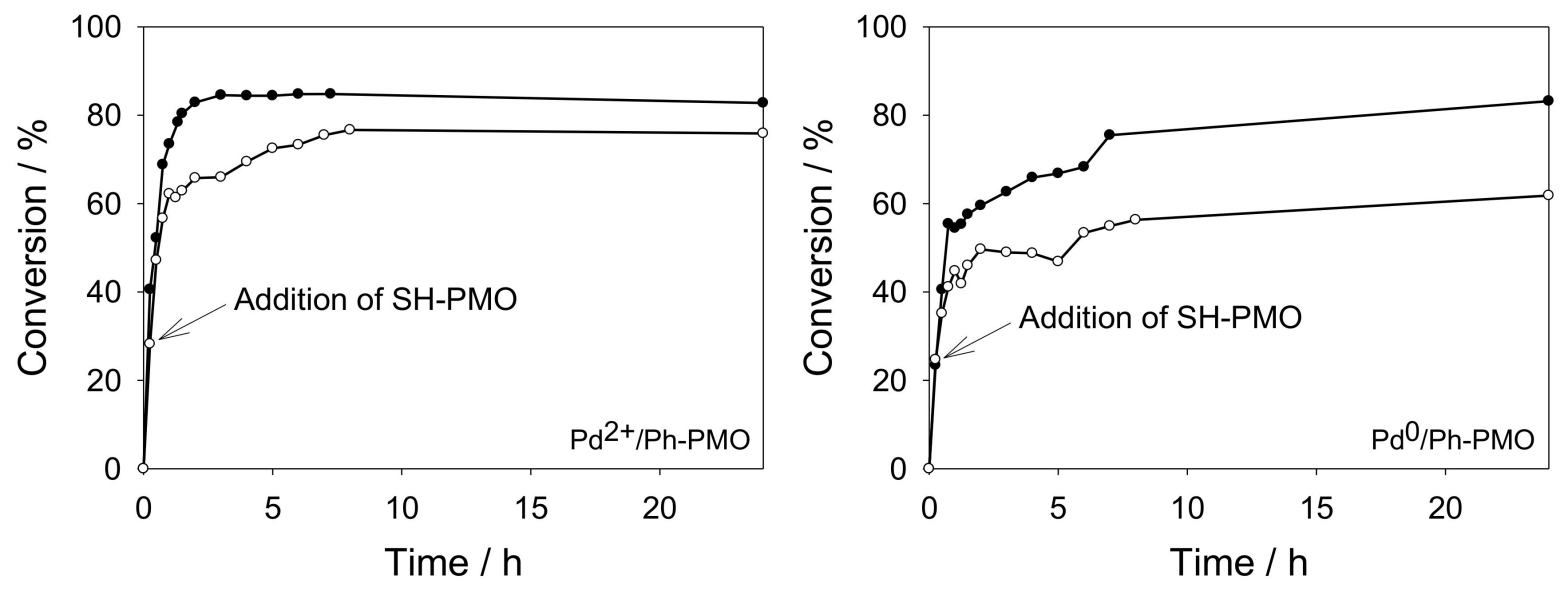
Catalyst poisoning test using Pd^2+^/Ph-PMO (left) and Pd^0^/Ph-PMO (right) as catalysts. SH-PMO material was added after 15 min reaction.

**Figure 7:**
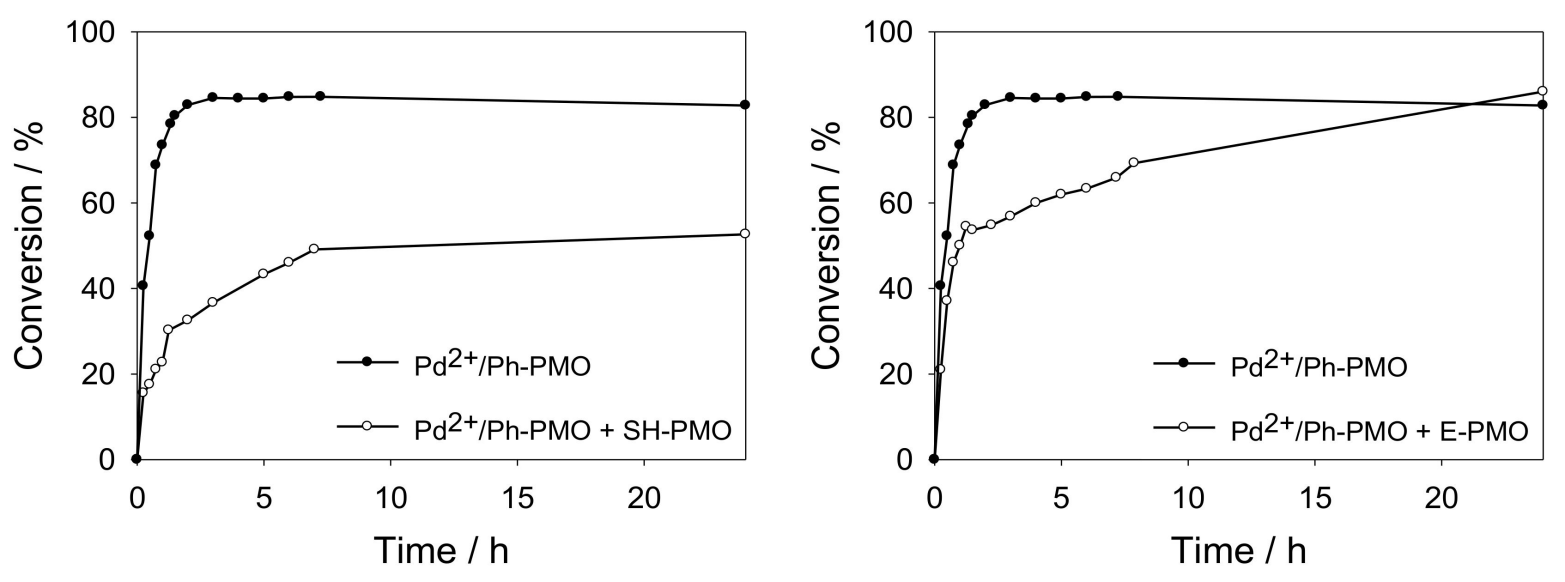
Catalyst poisoning tests using Pd^2+^/Ph-PMO as catalyst. SH-PMO (left) or E-PMO (right) materials were added at the beginning of the reaction.

A Pd scavenger added in a huge excess could block the active species and so stop the reaction. In view of the absence of activiy in Pd-SH-PMO, material SH-PMO was used as Pd scavenger to confirm the reaction mechanism. Figure 6 shows the effect of adding this material on the catalytic activity of Pd^2+^ or Pd^0^ supported on Ph-PMO after 15 min. The conversion to biphenyl decreased but the reaction seemed not to stop immediately. To confirm these results, Pd^2+^/Ph-PMO was used as catalyst and SH-PMO was added at the beginning of the reaction (Figure 7, left). The reaction rate and the yield to biphenyl were drastically reduced. Similarly, an ethylene-bridged PMO (E-PMO) was also added at the beginning of the reaction in order to compare the influence of adding a mesoporous organosilica without S. Although the reaction rate is decreased with respect to the pristine reaction, the yield to biphenyl at 24 h was practically the same (Figure 7, right).

## 4. Discussion

**Scheme 2:**
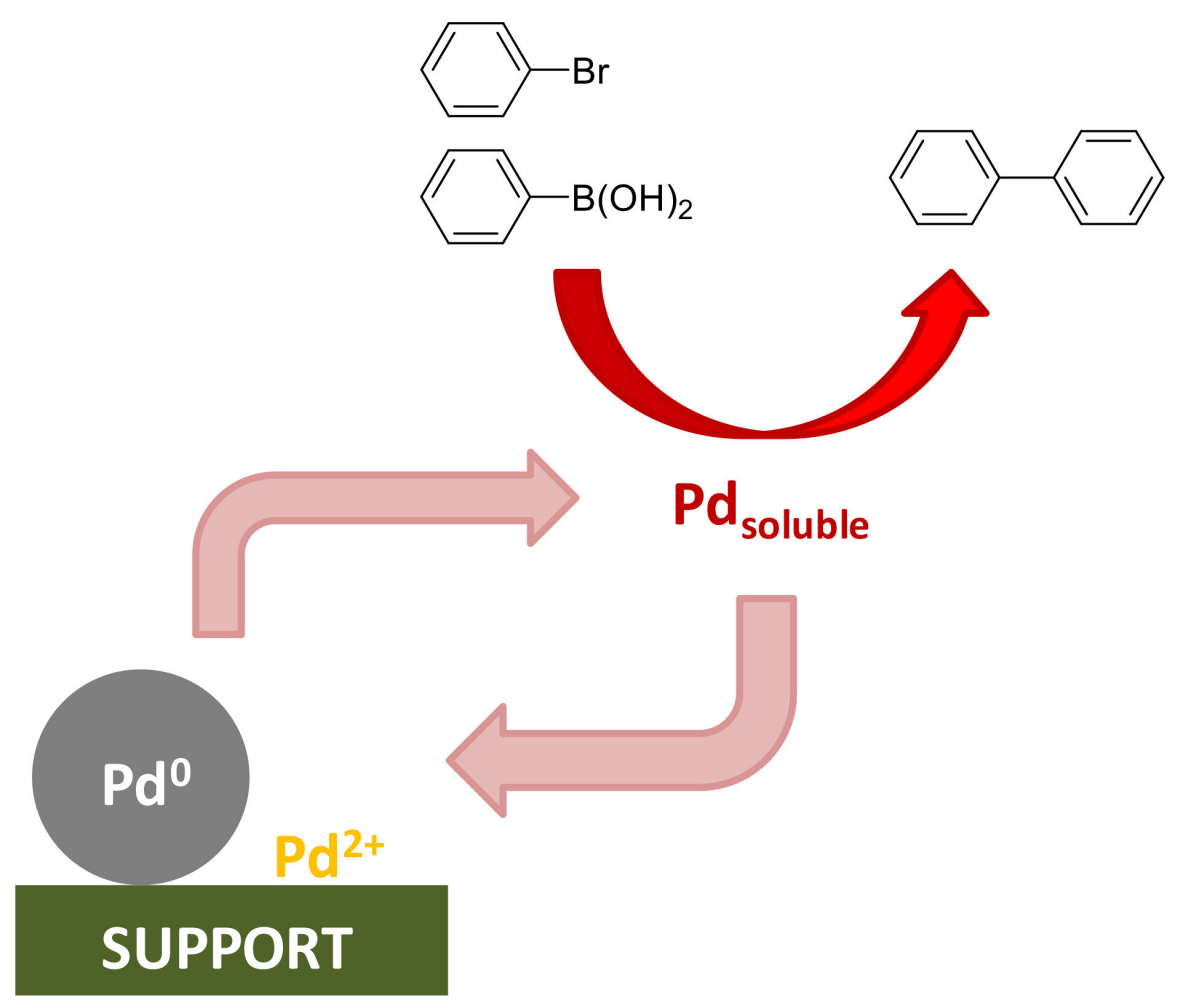
Schematic representation of the “release and catch” mechanism proposed for the palladium-catalyzed Suzuki cross-coupling reaction.

The results shown in the previous section can be explained according to the proposed “release and catch” mechanism (also called “release and recapture” or “release and capture”), in which supported palladium species become soluble and catalyze the Suzuki cross-coupling reaction, being redeposited again in the organosilica support (Scheme 2).

**Scheme 3:**
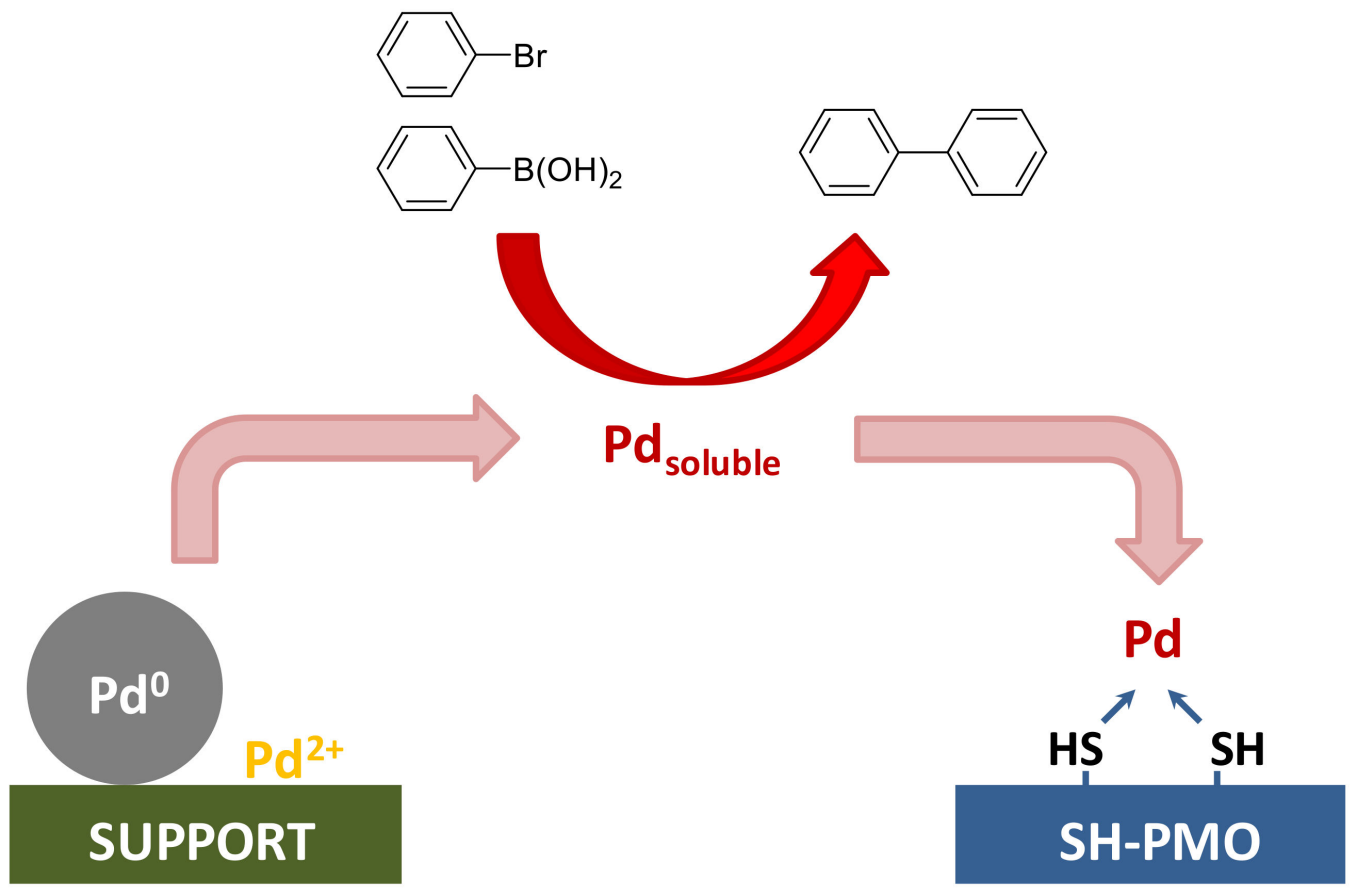
Schematic representation of the “release and catch” mechanism proposed for the palladium-catalyzed Suzuki cross-coupling reaction when SH-PMO is added to the reaction mixture, which acts as a palladium scavenger.

To be active, a catalyst must allow the exchange of Pd species with the solution as occurred for example with Pd-SH-E-PMO and Pd^2+^/Ph-PMO, among others. This is not the case for Pd-SH-PMO, where Pd^2+^ species are either so well coordinated to the support that they cannot escape into the solution or very efficiently recaptured by the support once released, so being unable to catalyze the reaction. When a second support is added to the reaction mixture (Figure 7, right), the reaction rate decreases since more Pd species will be on the surface of any of the solids even though the final conversion remains at the same level. However, the organosilica SH-PMO is so good Pd scavenger that when added to the reaction mixture (Figure 7, left), the reaction can only progress at the first stages and later stops since all Pd species have been retained on its surface thiol groups (Scheme 3).

Owing to the high toxicity of palladium in humans, the presence of residual palladium in the organic products is a matter of concern, particularly in the case of pharmaceutical compounds, which are generally synthesized without the benefit of chromatographic purification due to scalability reasons [27,28,53]. The results obtained showed that thiol-functionalized periodic mesoporous organosilica, synthesized using only 1-thiol-1,2-bis(triethoxysilyl)ethane as precursor, is a promising palladium scavenger with potential applications in the pharmaceutical industry.

## 5. Conclusions

Thiol-functionalized periodic mesoporous organosilicas with well-ordered mesoporous structures, high surface areas and narrow pore size distributions were synthesized using the novel precursor 1-thiol-1,2-bis(triethoxysilyl)ethane, alone or mixed with 1,2-bis(triethoxysilyl)ethane. After the incorporation of palladium, these materials, as well as palladium-supported periodic mesoporous (organo)silicas, were used as catalysts in the Suzuki cross-coupling reaction of bromobenzene and phenylboronic acid, showing important differences between them.

The use of different heterogeneity tests, such as hot filtration test and poisoning experiments, provided a deep knowledge of the reaction mechanism and demonstrated that the reaction likely takes place in homogeneous solution following a “release and catch” mechanism. Thereby, catalytic palladium species are leached out of the materials, then they carry out the coupling reaction and are redeposited on the catalyst surface at the end of the catalytic cycle. It has been checked by poisoning tests that the thiol-functionalized periodic mesoporous organosilica, synthesized using only 1-thiol-1,2-bis(triethoxysilyl)ethane as precursor, was especially useful for the recapture of palladium from the solution. This could be used for the removal of metallic species at the end of the catalytic cycles and in this way, low palladium contamination of the reaction mixture and products would be guaranteed. This issue is of enormous importance in pharmaceutical industries, where the requirements of residual metals in products are very strict.

## Figures and Tables

**Table 1:** Physicochemical properties of PMOs, before and after the incorporation of palladium.

Material	a_0_ (Å) ^1^	BET Surface Area (m^2^ g^−1^) ^2^	Pore Volume (cm^3^ g^−1^) ^3^	Pore Diameter (Å) ^4^	Wall Thickness (Å) ^5^
SH-PMO	96	448	0.49	45	51
Pd-SH-PMO	87	202	0.22	36	51
Pd-SH-PMO(H_2_)	81	211	0.22	34	47
SH-E-PMO	102	637	0.61	66	36
Pd-SH-E-PMO	100	269	0.31	58	42
Pd-SH-E-PMO(H_2_)	92	291	0.32	54	38

^1^ Unit-cell dimension calculated from a_0_ = (2d_100_/√3); ^2^ BET specific surface area determined in the range of relative pressures from 0.05 to 0.15; ^3^ Single point adsorption total pore volume; ^4^ Pore diameter determined from the maximum of the pore size distribution; ^5^ Estimated from (a_0_—pore diameter).

**Table 2:** Elemental compositions of PMOs, before and after the incorporation of palladium.

Material	Composition (wt%) ^1^		C/S Atomic Ratio	Pd (wt%) ^2^	Pd Incorporated (%)	S/Pd Atomic Ratio	S Atoms /nm^2^
	C	S					
SH-PMO	16.90	13.89	3.25	-	-	-	5.8
Pd-SH-PMO	11.80	11.99	2.63	5.32	23.4	7.5	11.1
Pd-SH-PMO(H_2_)	11.02	12.05	2.44	4.47	19.6	9.0	10.7
SH-E-PMO	18.36	6.79	7.22	-	-	-	2.0
Pd-SH-E-PMO	15.77	5.70	7.39	2.34	22.84	8.1	4.0
Pd-SH-E-PMO(H_2_)	13.87	4.72	7.84	2.13	20.79	7.4	3.1

^1^ Determined by elemental analysis; ^2^ determined by atomic absorption spectroscopy (AAS).

## Supplementary Materials

The following are available online at https://www.mdpi.com/1996-1944/13/3/623/s1, Figure S1: XRD patterns of PMOs, before and after the incorporation of palladium, Figure S2: N_2_ adsorption-desorption isotherms of PMOs, before and after the incorporation of palladium, Figure S3: Pore size distributions of PMOs, before and after the incorporation of palladium, Figure S4: DRIFTS spectra of SH-PMO, SH-E-PMO and E-PMO materials, Figure S5: XPS spectra of Pd 3d, Table S1: Physicochemical properties of E-PMO, Pd-E-PMO and Pd-E-PMO(H_2_) materials.
