# Supplementary material for: Thiol-Functionalized Ethylene Periodic Mesoporous Organosilica as an Efficient Scavenger for Palladium: Confirming the Homogeneous Character of the Suzuki Reaction
Source: Materials (Basel). 2020 Jan 30;13(3):623. doi: 10.3390/ma13030623 (PMC7040716; doi:10.3390/ma13030623)
Supplement: Supplementary file 1 [file materials-13-00623-s001.pdf]

materials

MDPI

Supplementary Materials: Thiol-Functionalized Ethylene Periodic Mesoporous Organosilica as an Efficient Scavenger for Palladium: Confirming the Homogeneous Character of the Suzuki Reaction

María I. López *, Dolores Esquivel *, César Jiménez-Sanchidrián, Pascal Van Der Voort and Francisco J. Romero-Salguero

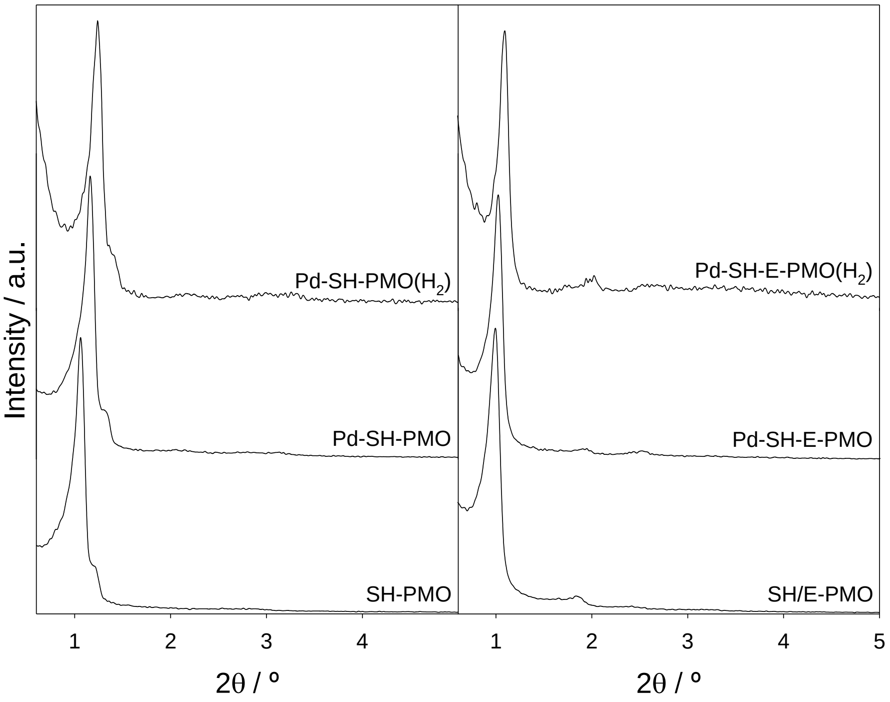

Figure S1. XRD patterns of PMOs, before and after the incorporation of palladium.

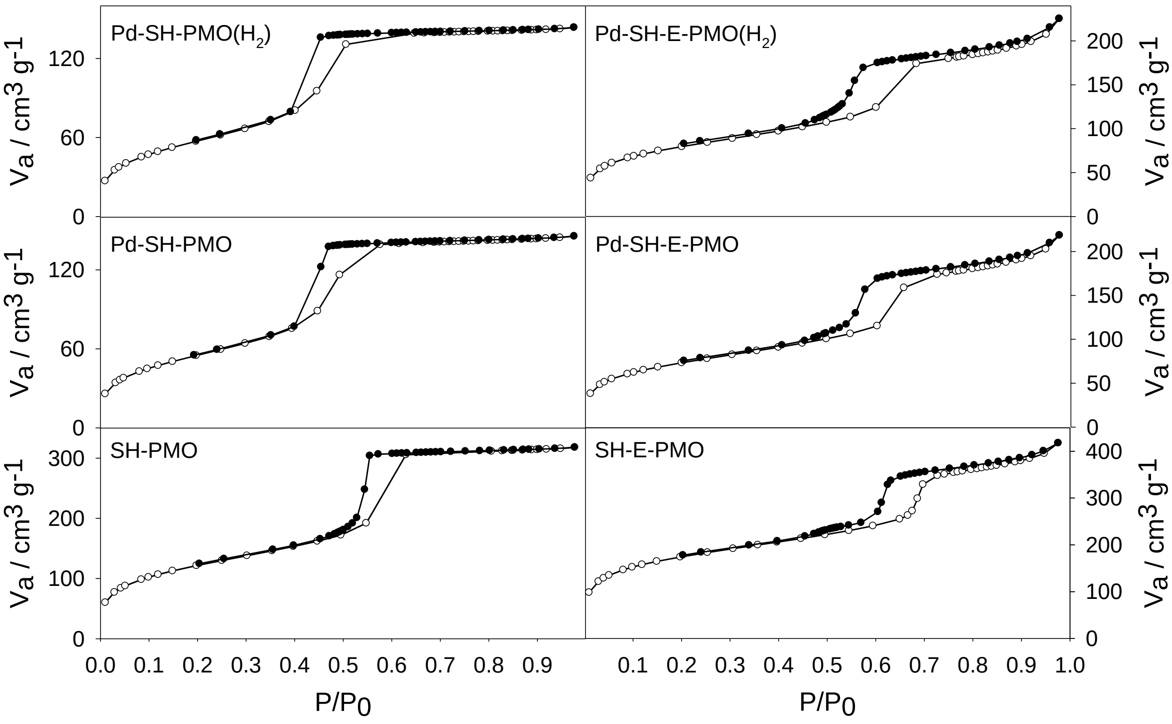

Figure S2. N_2 adsorption-desorption isotherms of PMOs, before and after the incorporation of palladium.

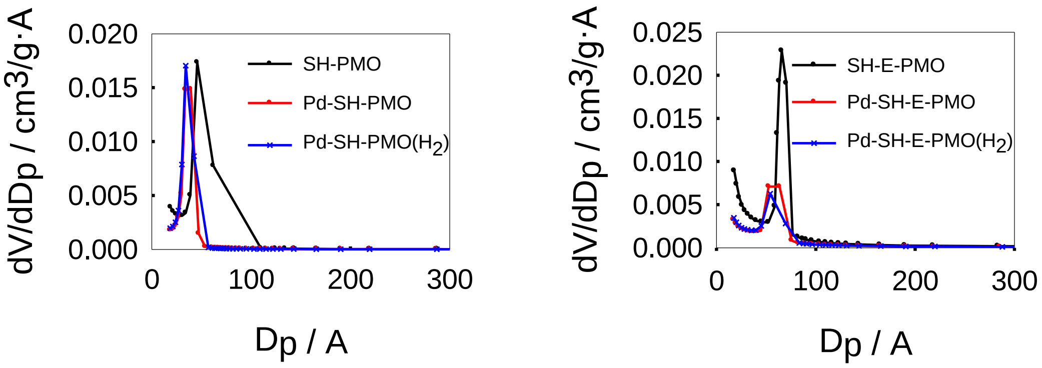

Figure S3. Pore size distributions of PMOs, before and after the incorporation of palladium.

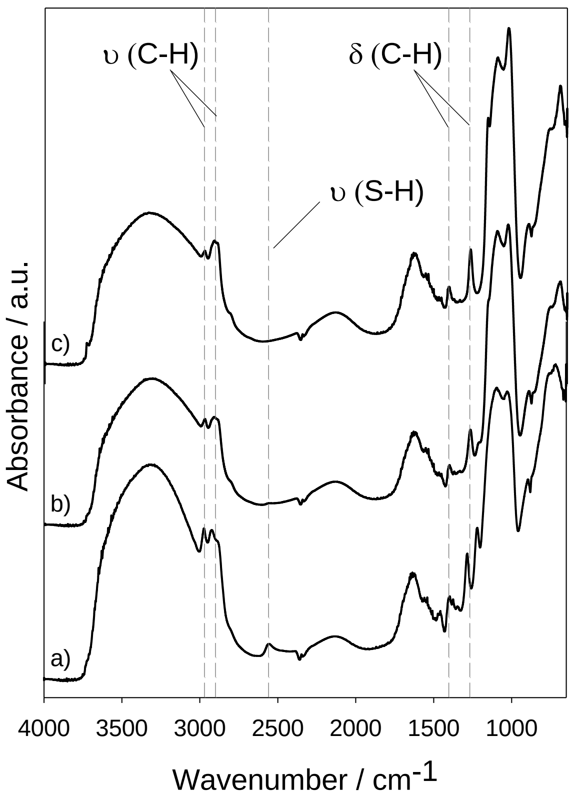

Figure S4. DRIFTS spectra: a) SH-PMO, b) SH-E-PMO, c) E-PMO.

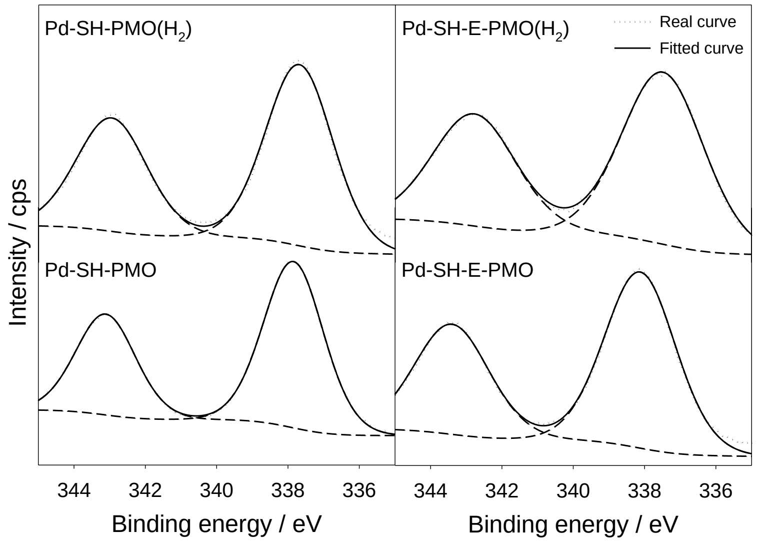

Figure S5. XPS spectra of Pd 3*d*.

Table S1. Physicochemical properties of E-PMO, Pd-E-PMO and Pd-E-PMO(H_2).

Material	a_0 (Å) [1]	BET surface area (m^2 g^{-1}) [2]	Pore volume (cm^3 g^{-1}) [3]	Pore diameter (Å) [4]	Wall thickness (Å) [5]
E-PMO	116	738	0.81	72	44
Pd-E-PMO	115	752	0.83	72	43
Pd-E-PMO(H_2)	103	579	0.69	69	34

[1] Unit-cell dimension calculated from $a_0 = (2d_{100}/\sqrt{3})$; [2] BET specific surface area determined in the range of relative pressures from 0.05 to 0.15; [3] Single point adsorption total pore volume of pores; [4] Adsorption average pore diameter determined from the pore size distribution; [5] Estimated from (a_0 – pore diameter).

© 2020 by the authors. Licensee MDPI, Basel, Switzerland. This article is an open access article distributed under the terms and conditions of the Creative Commons Attribution (CC BY) license (http://creativecommons.org/licenses/by/4.0/).
